# CST, an Herbal Formula, Exerts Anti-Obesity Effects through Brain-Gut-Adipose Tissue Axis Modulation in High-Fat Diet Fed Mice

**DOI:** 10.3390/molecules21111522

**Published:** 2016-11-11

**Authors:** AbuZar Ansari, Shambhunath Bose, Mukesh Kumar Yadav, Jing-Hua Wang, Yun-Kyung Song, Seong-Gyu Ko, Hojun Kim

**Affiliations:** 1 Department of Rehabilitation Medicine of Korean Medicine, Dongguk University, 814-Siksa-dong, Goyang, Gyeonggi-do 10326, South Korea; abu.zar.0313@outlook.com (A.A.); ewccwang@gmail.com (J.-H.W.); 2 NosQuest, 463-400 USPACE 1A-1103, Daewang Pangyoro 660, Bundanggu, Seongnamsi, Gyeonggi-do 13494, South Korea; shambose@yahoo.com; 3 Department of Otorhinolaryngology Head and Neck Surgery & Institute for Medical Device Clinical Trials, College of Medicine, Korea University, 148 Gurodong-ro, Guro-gu, Seoul 08308, South Korea; mukiyadav@gmail.com; 4 Department of Korean Rehabilitation Medicine, College of Korean Medicine, Gachon University, Incheon 22318, South Korea; lyricsong@naver.com; 5 Department of Preventive Medicine, College of Korean Medicine, Kyunghee University, Seoul 02453, South Korea; epiko@khu.ac.kr

**Keywords:** herb, *chowiseungcheng-tang*, obesity, neuropeptide, adipokine, gut microbiota, brain-gut-adipose tissue axis

## Abstract

The brain, gut, and adipose tissue interact to control metabolic pathways, and impairment in the brain-gut-adipose axis can lead to metabolic disorders, including obesity. *Chowiseungcheng-tang* (CST), a herbal formulation, is frequently used to treat metabolic disorders. Here, we investigated the anti-obesity effect of CST and its link with brain-gut-adipose axis using C57BL/6J mice as a model. The animals were provided with a normal research diet (NRD) or high-fat diet (HFD) in absence or presence of CST or orlistat (ORL) for 12 weeks. CST had a significant anti-obesity effect on a number of vital metabolic and obesity-related parameters in HFD-fed mice. CST significantly decreased the expression levels of genes encoding obesity-promoting neuropeptides (agouti-related peptide, neuropeptide Y), and increased the mRNA levels of obesity-suppressing neuropeptides (proopiomelanocortin, cocaine-and amphetamine-regulated transcript) in the hypothalamus. CST also effectively decreased the expression level of gene encoding obesity-promoting adipokine (retinol-binding protein-4) and increased the mRNA level of obesity-suppressing adipokine (adiponectin) in visceral adipose tissue (VAT). Additionally, CST altered the gut microbial composition in HFD groups, a phenomenon strongly associated with key metabolic parameters, neuropeptides, and adipokines. Our findings reveal that the anti-obesity impact of CST is mediated through modulation of metabolism-related neuropeptides, adipokines, and gut microbial composition.

## 1. Introduction

Obesity is a major global public health problem, closely associated with the onset and development of other diseases and disorders such as chronic inflammation, type 2 diabetes, insulin resistance, cardiovascular disease, neurodegenerative disorder, cancer as well as aging [1]. The major cause of obesity is thought to be an imbalance between energy intake and energy expenditure. Even a small imbalance between these components can lead to excessive adipose tissue accumulation [2], a key clinical manifestation of obesity. Although genetic and epigenetic factors are major determinants of obesity [3], accumulating evidence indicates that the gut microbiota vital in energy homeostasis also significantly influence obesity and the related metabolic disorders [4]. Since, diet has a profound impact on the gut microbial composition [5], it follows that gut microbiota act significantly in the modulation of diet-induced obesity.

The crosstalk between the organs and energy homeostasis regulation is crucial to sustain normal physiological functions. For instance, the inter-organ communication among the brain, gut, and adipose tissue, collectively termed the ‘brain-gut-adipose tissue axis’ is hypothesized as crucial in controlling several metabolic functions [6,7]. In an obese state, inter-organ communication is significantly disordered, which contributes to the changes in energy intake and energy expenditure, facilitates lipid deposition, and induces insulin resistance. Brain-derived neuropeptides influence the bidirectional communication between the components of brain-gut axis [8], while adipocyte-derived adipokines are integrally involved in energy homeostasis, neuroendocrine and immune functions [9]. Notably, alteration of the intestinal microbiota influences the profile of these neuropeptides and adipokines [10,11,12], stressing the importance of gut microbial environment in energy homeostasis and effect on obesity.

CST, a traditional herbal formula, is commonly used to treat a number of diseases including dementia [13], rheumatoid arthritis [14] and depression [15]. CST exerts beneficial effects on adiposity [16], obesity and hyperlipidemia [17], lipid metabolism, oxidation and inflammatory reflex [18]. Although the anti-obesity effect of CST has been documented, the detailed molecular mechanisms underlying this effect, especially in relation to neuropeptides, adipokines and abundance of gut microbiota have not been described. We hypothesize that CST exerts the anti-obesity effect through modulation of neuropeptides and adipokines and restoring the balance in the abundance of gut microbial population. To test our hypothesis, we examined the anti-obesity effect of CST in HFD-fed mice and further investigated the molecular mechanisms of action of CST on the obesity-related neuropeptide and adipokines and evaluated the ability of this herbal formulation to restore the gut microbial balance.

## 2. Results

### 2.1. CST Decreased Body, Liver, Adipose Tissue Weights and Food Efficiency Ratio in HFD-Fed Mice

**Figure 1:**
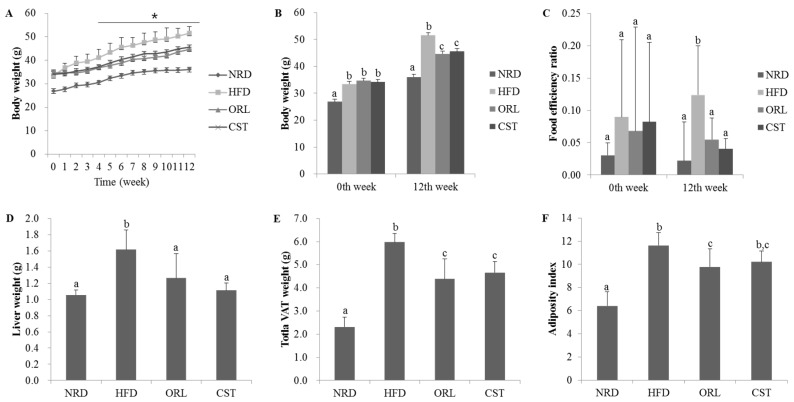
**Effect of CST on the body weight, food efficiency ratio, liver weight, and visceral adiposity in HFD-fed mice:** (**A**) Body weight of mice during entire experimental period (0–12 weeks); (**B**) Body weight of animals at 0th and 12th weeks of treatments; (**C**) Food efficiency ratio, (**D**) Liver weight; (**E**) Visceral adipose tissue weight and (**F**) Adiposity index at 12th week of treatment. Data represented the mean ± SD (*n* = 6). Different letters above bars indicate significant difference from each other at *p* < 0.05 as determined by one-way ANOVA with Least Significant Difference (LSD) post hoc test. * indicates statistical significance compared with HFD group at *p* < 0.05.

There were no significant differences in the initial (0th week) body weight among the HFD, ORL and CST groups. However, from the 3rd week onwards to 12th week, significant (*p* < 0.05) differences in the body weight were observed among the HFD, ORL and CST groups (Figure 1A). Notably, at 12th week, in addition to the body weight, liver weight, food efficiency ratio, weight of the total visceral adipose tissues (VAT; weight of adipose tissues of kidney, testis, and intestine in total), and visceral adiposity index of HFD-fed mice were found to significantly (*p* < 0.05) higher compared to those of NRD mice. However, all of these parameters significantly (*p* < 0.05) decreased in HFD-fed mice when treated with ORL. Except for the adiposity index, similar kinds of effects in HFD-fed mice were also shown by CST (Figure 1B–G).

### 2.2. CST Markedly Attenuated Blood Glucose and Insulin Levels and Moderately Decreased AUC of Blood Glucose in HFD-Fed Mice

**Figure 2:**
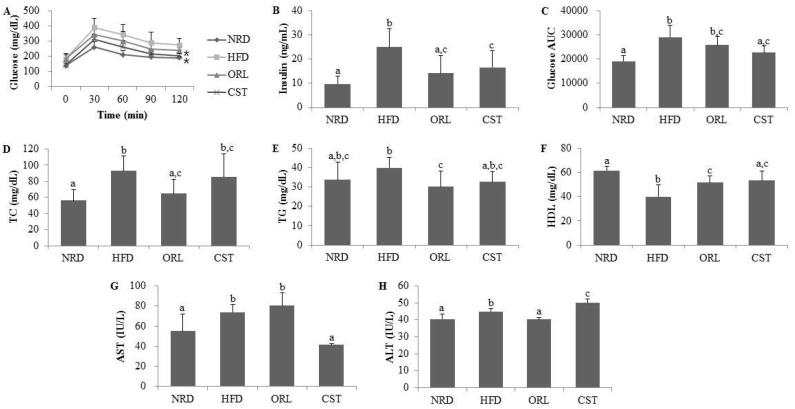
**Impact of CST on the glucose tolerance, insulin and other vital metabolic parameters:** (**A**) Blood glucose level; (**B**) Insulin level; and (**C**) Area under the curve (AUC) in oral glucose tolerance test (OGTT); (**D**) Total cholesterol (TC); (**E**) Triglyceride (TG); (**F**) High-density lipoprotein (HDL); (**G**) Aspartate transaminase (AST); and (**H**) Alanine transaminase (ALT). Data represented the mean ± SD (*n* = 6). Different letters above bars indicate significant difference from each other at *p* < 0.05 as determined by one-way ANOVA with Least Significant Difference (LSD) post hoc test. * indicates statistical significance compared with HFD group at *p* < 0.05.

The OGTT profile showed significantly (*p* < 0.05) increased levels (Figure 2A) and AUC (Figure 2C) of blood glucose in the HFD-fed mice compared with NRD mice. However, exposure of HFD-fed mice to CST significantly (*p* < 0.05) suppressed the blood glucose level (Figure 2A), and decreased the AUC, although chance could not be ruled out (Figure 2C). The fasting serum insulin level was also significantly (*p* < 0.05) higher in HFD-fed mice compared with NRD mice (Figure 2B). However, a significant reduction in the level of serum insulin was observed in the HFD-fed mice due to an exposure to ORL and CST.

### 2.3. CST Ameliorated the Lipid Profile of HFD-Fed Mice and Influenced the Serum AST and ALT Activities

Significantly (*p* < 0.05) higher levels of serum TC, but not TG, were observed in the HFD-fed mice compared with NRD mice (Figure 2D,E). However, the TG level of HFD-fed mice decreased significantly (*p* < 0.05) when treated with either ORL or CST (Figure 2E). Moreover, treatment of HFD-fed mice with ORL significantly (*p* < 0.05) lowered the serum TC level (Figure 2D). A reduction in the serum TC level in the HFD-fed mice due to an exposure to CST failed to reach statistical significance. The serum HDL level was significantly (*p* < 0.05) lower in HFD-fed mice vs. NRD mice (Figure 2F). However, the HDL level of HFD-fed mice was significantly (*p* < 0.05) elevated when exposed to either CST or ORL. The activities of liver enzymes AST and ALT were significantly (*p* < 0.05) higher in the sera of HFD-fed mice compared with that of NRD mice (Figure 2G,H). The AST activities of HFD-fed mice increased slightly although chance could not be ruled out and ALT significantly (*p* < 0.05) decreased when treated with ORL (Figure 2G,H). In contrast, exposure of HFD-fed mice to CST resulted in a significant (*p* < 0.05) reduction in AST activity (Figure 2G) but a significant increase in ALT activity (Figure 2H). Further studies are needed to find the exact basis of this differential regulation of AST and ALT activities by CST in the HFD-fed mice. Similar differential responses of AST and ALT was observed in rats when treated with extract of *Cannabis sativa*, an herb long used as psychoactive medicine [19]. CST extract possesses antioxidant activity [18], and a clinical trial evaluating the effect of antioxidant supplements non-alcoholic fatty liver disease and/or steatohepatitis [20], found a significant (*p* = 0.004) reduction in AST activity along with significantly increased ALT activity (*p* < 0.00001) compared to placebo-treated subjects.

### 2.4. CST Decreased HFD-Induced Hepatosteatosis and Adiposity

**Figure 3:**
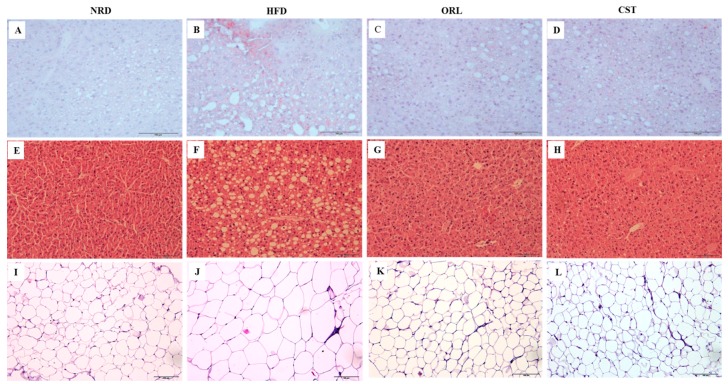
**Histopathology of liver and adipose tissues:** Frozen sections of the liver tissues stained with oil-O-red (**A**–**D**) and counter stained with hematoxylin and eosin (H & E) (**E**–**H**); Paraffin sections of fat tissues stained with H & E (**I**–**L**). The histological examinations of the tissue sections were performed under light microscopy with 200× magnification (scale bar 100 μm).

The histological analysis of frozen liver tissue sections stained with oil-O-red revealed minimal fat droplets in the hepatic tissues of NRD, but high abundance in HFD-fed mice (Figure 3A,B). However, a marked reduction in the hepatic lipid accumulation was observed in the HFD-fed mice in response to the treatment with ORL or CST (Figure 3C,D). Further, histological evaluation of hematoxylin and eosin-stained paraffin tissue sections of NRD mice revealed normal lobular architecture of the liver with negligible appearance of large vacuoles (Figure 3E). In contrast, the liver of HFD-fed mice exhibited an aberrant histological structure characterized by abundant large vacuoles indicating increased hepatosteatosis and ballooning (Figure 3F). However, minimal small vacuoles were observed in HFD-fed mice when exposed to ORL or CST (Figure 3G,H). Moreover, the histology of hematoxylin and eosin-stained paraffin sections of adipose tissues showed the appearance of small adipocytes in the NRD, but large in HFD-fed mice (Figure 3I,J). Similar to NRD mice, only small adipocytes were observed in the adipose tissues of HFD-fed mice in response to treatment with ORL or CST (Figure 3K,L).

### 2.5. Impact of CST on the Expression of Neuropeptides and Adipokines in HFD-Fed Mice

**Figure 4:**
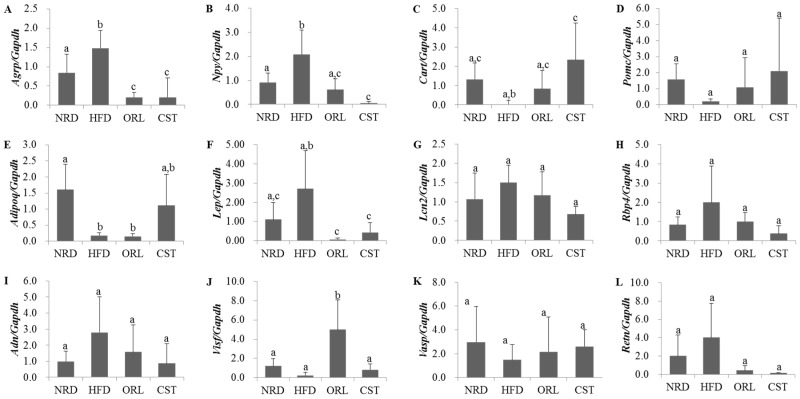
**Expression levels of neuropeptides and adipokines:** (**A**–**D**) Gene expression levels of neuropeptides in hypothalamus (*n* = 6); (**E**–**L**) Gene expression levels of adipokines in adipose tissues (*n* = 3). Levels of mRNAs were measured by qRT-PCR and the fold values were normalized using the house keeping gene *Gapdh*. Data represented as mean ± SD (*n* = 3–6). Different letters above bars indicate significant difference from each other at *p* < 0.05 as determined by one-way ANOVA with Least Significant Difference (LSD) post hoc test.

We examined the levels of expression of obesity-related neuropeptides in the hypothalamus and adipokines in adipose tissue using qRT-PCR. The expression of genes encoding obesity-promoting neuropeptides *Agrp* and *Npy* were significantly (*p* < 0.05) up-regulated in HFD-fed mice compared with NRD mice (Figure 4A,B). However, the expression of both of these genes in HFD-fed mice were significantly (*p* < 0.05) down-regulated when treated with either CST or ORL. In contrast, the mRNA level of the obesity-suppressing neuropeptide *Cart* in HFD-fed mice was lower than the NRD mice, although chance could not be ruled out (Figure 4C); it increased significantly (*p* < 0.05) when treated with CST, but not ORL. The gene expression of *Pomc* did not differ significantly between HFD-fed and NRD mice (Figure 4D), while the level of adipokine *Adipoq* mRNA was significantly down-regulated in HFD group vs. NRD group (Figure 4E). The levels of expression of both *Pomc* and *Adipoq* genes in HFD-fed mice did not change significantly when treated with either CST or ORL. The expression of *Lep* gene in the HFD group was higher versus the NRD group, although chance could not be ruled out, but reduced significantly when exposed to either ORL or CST (Figure 4F). The mRNA levels of *Lipo2*, *Rbp4*, *Adn* and *Retn* adipokines in HFD-fed mice were higher compared with NRD mice, and decreased when treated with either CST or ORL (Figure 4G–I,L), although chance could not be ruled out in either case. In contrast, the expressions of both *Visf* and *Vasp* in HFD-fed mice were lower than in NRD mice (Figure 4J,K), although chance could not be ruled out. The mRNA level of *Vasp* in HFD group increased when treated with either ORL or CST, although chance could not be ruled out. The expression of *Visf* in HFD-fed mice increased significantly and insignificantly when exposed to ORL but not when exposed to CST.

### 2.6. CST Modulated the Relative Abundance (RA) of Putative Beneficial Gut Microbiota in HFD-Fed Mice

**Figure 5:**
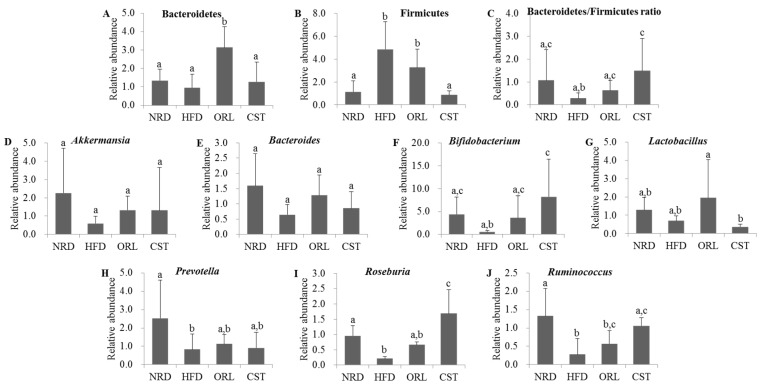
**Relative abundance of related microbes in mice fecal samples:** (**A**–**C**) The relative abundance of phyla; and (**D**–**J**) the relative abundance of genus of microbiota in the stool samples of mice as reflected by microbial 16sRNA gene expression. The results are expressed as normalized fold values relative to the normal group. Data represented as mean ± SD (*n* = 5). Different letters above bars indicate significant difference from each other at *p* < 0.05 as determined by one-way ANOVA with Least Significant Difference (LSD) post hoc test.

We quantified the RA of fecal microbiota of 12th-week samples at the phyla or genus levels using qRT-PCR. The exposure of HFD-fed mice to ORL resulted in significant (*p* < 0.05) increases in RA of Bacteroidetes, and CST exposure resulted in slight RA increase although chance could not be ruled out (Figure 5A). In contrast, RA of Firmicutes in HFD group was decreased significantly (*p* < 0.05) upon treatment with CST, but not ORL (Figure 5B). Accordingly, the ratio of Bacteroidetes/Firmicutes was significantly (*p* < 0.05) higher in CST-, but not ORL-treated HFD-fed mice compared with HFD-fed mice (Figure 5C). The RA of *Akkermansia*, *Bacteroides, Lactobacillus* and *Prevotella* did not differ significantly between the HFD-fed mice and HFD-fed mice treated with ORL or CST groups (Figure 5D,E,G,H). The RA of *Bifidobacterium*, *Rosenburia* and *Ruminococcus* were significantly (*p* < 0.05) elevated in HFD-fed mice when treated with CST, but not ORL (Figure 5F,I,J).

### 2.7. CST-Mediated Modulation in Gut Microbial Composition Correlated with Improvements in Metabolic Parameters in HFD-Fed Mice

**Figure 6:**
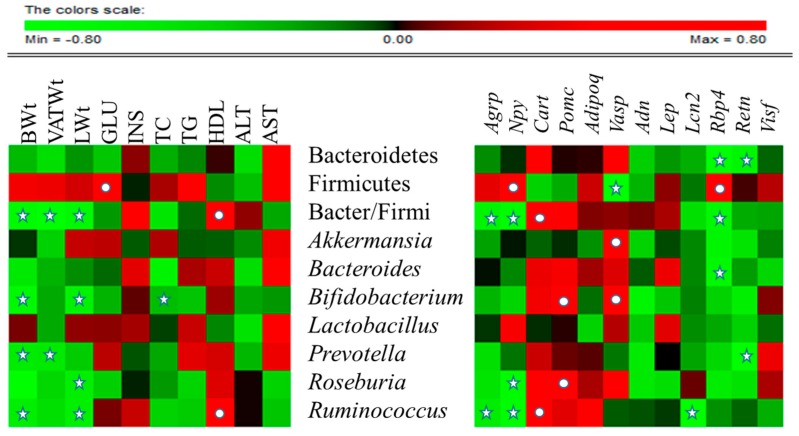
**Correlation between gut microbial relative abundance and host metabolic parameters, neuropeptides and adipokines:** Data of all experimental groups (except normal) were gathered and analyzed by SPSS software (17.0. version, Chicago, IL, USA) using Spearman’s rho calculated by Permut Matrix software (version 1.9.3 EN, Montpellier, France) for heatmap plots. As the colors scale shows, green color indicates a negative correlation, while red color denotes a positive correlation. The ★ symbol indicates statistically significant negative correlation (*p* < 0.05) and • symbol indicated statistically significant positive correlation (*p* < 0.05).

Association of the RA of gut microbiota with metabolic markers or expression levels of vital neuropeptides and adipokines were assessed in all HFD-fed experimental groups by Spearman rho (Figure 6). At the phylum level, Bacteriodetes was significantly negatively correlated with *Rbp4* (*p* < 0.05) and *Retn* (*p* < 0.05). 

Firmicutes was significantly negatively correlated with *Vasp* (*p* < 0.05) and positively correlated with blood glucose (*p* < 0.05), *Npy* (*p* < 0.05) and *Rbp4* (*p* < 0.05). The Bacteriodetes/Firmicutes ratio was significantly negatively correlated with body weight (*p* < 0.05), total VAT weight (*p* < 0.05), liver weight (*p* < 0.05), *Agrp* (*p* < 0.05), *Npy* (*p* < 0.05) and *Rbp4* (*p* < 0.05), and was significantly positively correlated with HDL, (*p* < 0.05) and *Cart* (*p* < 0.05). At genus level, *Akkermansia* was significantly positively correlated with *Vasp* (*p* < 0.05), and *Bacteroides* significantly negatively correlated with *Rbp4* (*p* < 0.05). *Bifidobacterium* exhibited significant negative correlation with body weight (*p* < 0.05), liver weight (*p* < 0.05), and total cholesterol, and significant positive correlation with *Pomc* (*p* < 0.05) and *Vasp* (*p* < 0.05). *Prevotella* was negatively correlated with body weight (*p* < 0.05), total VAT weight (*p* < 0.05) and *Retn* (*p* < 0.05). *Roseburia* was significantly negatively correlated with liver weight (*p* < 0.05) and *Npy* (*p* < 0.05), and significantly positively correlationed with *Pomc* (*p* < 0.05). *Ruminococcus* revealed significant negative correlation with body weight (*p* < 0.05), liver weight (*p* < 0.05), *Agpr* (*p* < 0.05), *Npy* (*p* < 0.05), and *Lcn2* (*p* < 0.05), and significant positive correlation with HDL (*p* < 0.05) and *Cart* (*p* < 0.05). In contrast, *Lactobacillus* was not significantly correlated with any of the parameters studied. Collectively these associations suggest that CST affects the gene expression of adipokines and neuropeptides and alters the RA of gut microbiota.

## 3. Discussion

Due to low side-effects, the use of herbal medicines in the treatment of obesity has increased recently. Indeed, many herbal medicines are very effective against obesity, especially at reducing body weight, fat weight and decreasing food intake, with minimal or no side-effects [21]. Very few studies have been carried out to understand the mechanisms of action of herbal medicines against obesity. The involvement of brain-gut-adipose tissue in particular has not been studied in detail. The function of this axis is regulated by the interaction among neuropeptides, adipokines, and gut microbiota. The hypothalamus is central in the mechanistic chain of the brain-gut-adipose tissue axis because it consists of receptors for neuropeptides as well as adipose tissue-derived adipokines, which ultimately communicate with the brain via neuronal inputs [22]. Notably, gut microbiota interact with food to generate a surplus of acetate, which promotes obesity [23], and this signal is transmitted via the vagal nerve, which connects the gut with the brain. In some cases, gut microbiota sends signals to the brain to eat more and promote weight gain [24]. Using HFD-induced mouse or rat obese models, CST affects lipid metabolism, oxidation, as well as inflammatory reflex and has been shown to possess anti-obesity and anti-hyperlipidemic activities [17]. However, the mode of action of CST, and its relation with the brain-gut-adipose tissue axis is not known. Here, we investigated the effect of CST on HFD-induced obese mice and elucidated the molecular mechanisms underlying the influence of CST on the brain-gut-adipose axis and the RA of gut microbiota, the two vital factors in the onset and development of obesity.

The gut microbial population influences the biochemical activity of the host by providing extra metabolic functions and controlling a variety of molecular aspects of cellular differentiation and gene expression through host-microbe interactions [25]. Furthermore, the gut microbiota serves as the sources of enzymes involved in the utilization of non-digestible carbohydrates and host-derived glycoconjugates, deconjugation and dehydroxylation in the bile acids, cholesterol reduction and biosynthesis of vitamins (K and B groups), isoprenoids and amino acids such as lysine and threonine [25]. In general, the microbial fermentation of carbohydrates in the gut typically produces acetate, propionate, butyrate, and lactate, which are the members of specific short chain fatty acids (SCFAs) [26]. The relative abundance SCFAs may play an important role in determining a specific hot response. Notably, this metabolic collaboration relies on the presence of a particular genus of bacteria since all substrates (nutrients) are not equally converted into SCFAs through carbohydrate fermentation. Moreover, not all the SCFAs produce similar metabolic impact. For instance, in theory, acetate serves as a cholesterol of fatty acid precursor, while propionate is involved in the gluconeogenic pathway in the liver and the gut, but at the same time may also neutralize lipogenesis from acetate or glucose in the liver [26].

Several lines of evidence support HFD as a potent inducer of weight and fat gain [27], accompanied by alteration in gut microbiota [28]. More specifically, obesity is found to be associated with phylum and group-specific changes in the microbiota [25]. The gut microbial population is dominated by two main bacterial phyla types: Bacteroidetes and Firmicutes [29]. Based on accumulating evidence, it is conceivable that a decrease in the relative proportion of Bacteroidetes as well as a reduction in the ratio of Bacteroidetes to Firmicutes is associated with an increase in body weight [30,31,32]. This agrees with those investigations reporting a decrease in Bacteroidetes in obesity and an increase in Firmicutes in response to HFD [33,34]. In the present study, we observed an increase in the gut population of Bacteroidetes and a significant decreased in Firmicutes in the HFD-fed mice treated with CST. The abundance of Firmicutes in the HFD-fed experimental groups was significantly positively correlated with the blood glucose level measured during an oral glucose tolerance test. A higher ratio of the RA of Bacteroidetes/Firmicutes was observed in the HFD-fed CST treated mice compared to HFD-fed mice, and this ratio in all HFD-fed experimental groups maintained a significant negative correlation with the body, adipose tissue and liver weight.

Notably, a drastic reduction in the gut population of *Bifidobacterium* was seen in the mice when fed with HFD, although this change did not reach statistical significance. This agrees with study where HFD-fed mice exhibited obesity phenotype accompanied with a reduction in the intestinal population of *Bifidobacterium* sp. and the onset of metabolic endotoxemia, which in a chronic state induced obesity, diabetes, and liver insulin resistance [35]. A significantly lower number of *Bifidobacteria* has been reported in obese subjects in comparison to lean volunteers [36]. Furthermore, an increase in the population of *Bifidobacterium* exerts anti-obesity and lipid-lowering effects against high fat [37] and facilitates uptake of cholesterol [38], in keeping with the action of *Bifidobacterium breve* strain B-3 supplementation as a suppressor of obesity in a mouse model of HFD-induced obesity [39]. In our study, *Bifidobacterium* sp. is significantly negatively correlated with body and liver weight, as well as total cholesterol in the entire HFD-fed experimental groups, supporting the anti-obesity effect of this bacterial species. Notably, a significant increase in the abundance of *Bifidobacterium* sp. was observed in the HFD-fed mice when treated with CST. Based on this, it is conceivable that an increase of gut *Bifidobacterium* may be pivotal in the anti-obesity effect of CST on the HFD-fed mice, although no definite conclusion can be made without further studies.

We observed a significant reduction in the gut population of *Prevotella* and *Roseburia* in the mice in response to feeding HFD. This agrees with studies revealing decreased abundance of *Bacteroides*, *Prevotella* and *Roseburia* in mice in response to HFD feeding and in human subjects due to obesity [36,40,41]. Moreover, in the HFD-fed experimental groups, we observed significant negative correlations between the abundance of gut *Prevotella* and body and VAT weight as well as a significant negative correlation between the gut population of *Roseburia* and liver weight. Furet et al. (2010) [41] reported that body weight, BMI, body fat mass, and serum leptin concentrations of obese subjects were negatively correlated with the counts of *Bacteroides* and *Prevotella* groups in the fecal samples examined after gastric surgery. Likewise, significant negative correlations were found between the caecal content of *Roseburia* and body weight gain, subcutaneous adipose tissue weight, feed efficiency, fasting glycemia, AUC of the glucose excursion following oral glucose load, hepatic triglycerides, and plasma total cholesterol in HFD-fed mice [40]. It was reported that wheat arabinoxylan (AX) treatment, which significantly decreased HFD-induced adiposity, body weight gain, serum and hepatic cholesterol accumulation, and insulin resistance in mice, restored the number of caecal bacterial population of *Bacteroides-Prevotella* and *Roseburia* that had decreased with HFD feeding of the animals [36]. Similarly, fungal chitin glucan fiber treatment, which significantly reduced HFD-induced body weight gain, fat mass development, fasting hyperglycemia, glucose intolerance, hepatic triglyceride accumulation and hypercholesterolemia in mice, restored the abundance of gut bacteria from Clostridia cluster XIVa including *Roseburia* sp. that had declined in response to HFD feeding [40]. Notably, in our study, non-significant and significant increases in the RA of *Prevotella* and *Roseburia*, respectively were seen in the HFD-fed mice when exposed to CST. This suggests that elevation of the above two bacterial genera may contribute to the anti-obesity impact of CST on the HFD-fed mice, although no definite conclusion can be drawn without further investigations.

The present study also revealed a significant decline in the gut population of *Runimococcus* in the mice upon HFD feeding. The population of this bacterial species in the HFD-fed groups demonstrated significant negative correlations with the body and liver weights and a significant positive correlation with HDL cholesterol. This is in contrast to reports stating that *Ruminococcus* facilitates the absorption of sugars by the cells [35], which might contribute to weight gain [36], although the relationship between weight loss or gain and *Ruminococuss* has not been adequately described [5]. In our study, the gut population of *Ruminococuss* sp. increased significantly in the HFD-fed mice when treated with CST. In summation, our results indicate that CST ameliorates HFD-induced obesity, at least in part, through modulation of gut microbial population, facilitating restoration of the RA of beneficial bacterial species and increase in the Bacteroidetes-to-Firmicutes ratio.

The communication between brain and gut is bidirectional, mediated through the interaction between gut and brain-derived hormones and neuropeptides [12]. It has been found that, gut microbes may interact with the enteric nervous system (ENS), afferent nerves (vagal sensory neurons, spinal sensory neurons and intrinsic primary afferent neurons (IPANs)), and the central nervous system (CNS) to modulate the production and/or release of neurotransmitters through direct or indirect actions on neurons [26]. A major function of the hypothalamus is to serve as the “appetite center” which controls appetite and satiety. The hypothalamus produces a number of hormones and neuropeptides and is enriched with receptors for non-brain derived hormones like insulin and adipose tissue-derived adipokines like leptin that are related to obesity [6,42]. The genes encoding hypothalamus-derived neuropeptides *Agrp* and *Npy* promote obesity while genes for *Cart* and *Pomc* suppress obesity [7]. *Agrp* and *Npy* stimulate food intake and promote weight gain [43], the major hallmarks of onset and development of obesity. Here, we observed significantly lower mRNA levels of *Agrp* and *Npy* as well as higher expression levels of *Cart* and *Pomc* genes (although we could not always rule out chance), in CST treated HFD-fed mice compared with HFD-fed mice. As mentioned above accumulating evidence indicate that an alteration in the gut microbial population influence the profile of the neuropeptides [10,11], further supporting the roles of gut microbiota in energy homeostasis and contribution towards the obesity. In the present study, correlations of the RA of gut microbiota with the expression levels of vital neuropeptides were studied in the HFD-fed experimental groups. We found a significant positive correlation between the *Npy* gene expression and gut population of obesity-promoting microbiota Firmicutes. This is in keeping with the ratio of Bacteroidetes/Firmicutes which maintained significant negative correlations with the expression of *Agrp* and *Npy* genes and a significant positive correlation with the mRNA level of *Cart*. *Rosenburia* showed a significant negative correlation with the *Npy* gene expression and a significant positive correlation with *Pomc* mRNA level. *Ruminococcus* on the other hand was significantly negatively correlated with the expression of *Argp* and *Npy* genes and significantly positively correlated with the *Cart* mRNA level. Considering all of the above, CST may be vital in the modulation of the RA of gut microbiota that influence the expression of neuropeptides linked to energy homeostasis and obesity.

Adipose tissue serves as the main site of lipid storage and also acts as an endocrine organ [44]. The cross-talk between adipose tissue and brain is regulated through an interaction between the adipose-tissue derived adipokines and their receptors present in the brain. Additionally, growing evidence suggests the importance of the gut microbiota in the host metabolism through a bidirectional axis of communication with the adipose tissue, which influences the onset and development of metabolic alterations associated with obesity [11]. A recent study observed that bacterial cell wall components could affect the onset of metabolic syndromes by mediating the secretion of adipokines from VAT [45]. *Lep*, an important and well-studied adipokine, binds to the leptin receptors in the hypothalamus and regulates feed intake and energy expenditure [46,47], and is directly related to obesity severity [48,49]. In clinical scenario, obese subjects usually have elevated serum leptin levels associated with increased hunger and reduced energy expenditure [25]. Moreover, augmented leptin levels could also trigger the production of pro-inflammatory t-helper 1-type cytokines and contribute to the inflammatory response associated with the obesity [25]. Like *Lep*, *Adipoq* also binds to its own receptors in the hypothalamus, but facilitates fatty acid oxidation and enhances insulin sensitivity [50]. Consistent with this, *Adipoq* expression is reduced in subjects suffering from obesity in association with insulin resistance or type 2 diabetes [51]. In the present study, we observed a significant down-regulation of *Lep* and an up-regulation of *Adipoq* gene expressions that did not reach significance in the HFD-fed mice upon treatment with CST. *Vasp*, another adipokine, improves insulin sensitivity and suppresses the production of *Retn*, *Lep*, and *TNF-α* [52]. Here, we observed that treatment of HFD-fed mice with CST caused a definite increase, although the rule of chance could not be excluded, in the expression of *Vasp* which is significantly negatively correlated with Firmicutes and significantly positively correlated with *Akkermansia* and *Bifidobacterium*. On the other hand, *lcn2*, a novel proinflammatory adipokine is associated with obesity, insulin resistance, and hyperglycemia in humans [53]. Here, our results showed a definite decrease in the mRNA level of *Lcn2* in HFD-fed mice in response to an exposure to CST, although the change did not reach statistical significance. A significant negative correlation was observed between *Lcn2* expression and gut *Rumincoccus* abundance, in keeping with the negative association of this bacterial species with the obesity parameters as discussed above. The *Retn* is a cysteine-rich adipocyte-derived peptide hormone whose over-production is responsible for the development of insulin resistance [54]. Studies on *Retn* in relation to obesity reveal higher serum *Retn* levels in obese subjects than lean subjects [54]. CST treatment caused a definite decrease, although we failed to reject the null hypothesis of no change, in the expression of *Retn* gene in the HFD-fed mice, signifying a suppressive effect of CST on insulin resistance.

For recently discovered adipokines *Visf* and *Rbp4*, no clear targets in the brain have been identified so far, and no information regarding their roles in the brain-adipose tissue communication has been documented. However, *Rbp4* promotes insulin resistance and lack of *Rbp4* improves glucose homeostasis [55]. Here, we observed a definite decrease in the expression of *Rbp4* gene in CST-treated HFD-fed mice compared with HFD-fed mice, although the rule of chance could not be excluded. Significant negative correlations between the *Rbp4* expression and the gut abundance of Bacteroidestes and *Bacteroides* as well as Bacteroidetes/Firmicutes ratio were noticed. In agreement, a significant positive correlation between the mRNA level of *Rbp4* and gut population of Firmicutes was observed. These results imply that CST suppresses the expression of *Rbp4* where CST-modulated RA of the above mentioned gut microbiota may play a crucial role.

**Figure 7:**
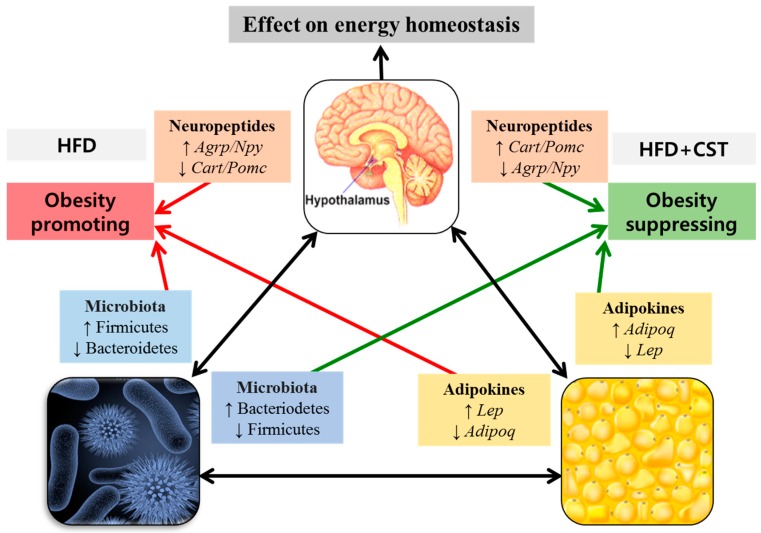
The possible involvement of gut microbial composition in the inter-communication among the vital components of brain-gut-adipose tissue axis associated with host metabolism. The probable interactions between and among the brain-derived neuropeptides, adipose tissue-derived adipokines and the modulated gut microbial communities resulting in the promotion or suppression of obesity are shown.

Collectively, our findings suggest that CST combats HFD-induced obesity, at least in part, thorough its beneficial impact on the gene expression of brain-derived neuropeptides and adipokines of fat tissues, the key players in energy homeostasis and metabolism. Probably, these effects of CST are mediated through an improvement in the balance of gut microbial ecology (Figure 7).

In conclusion, functional synchronization between the components in the axis of metabolic pathways is requisite to maintain the normal physiological processes. In case of brain-gut-adipose tissues axis, synchronization is achieved through interaction between and among the gut-secreted hormones or peptides, intestinal microbiota, adipose tissue-derived adipokines, and brain derived-neuropeptides. Our findings indicate that CST treatment exerts an anti-obesity effect on HDF-induced obesity. This beneficial impact of CST is mediated through the modulation of obesity-related neuropeptides and adipokines of the brain-gut-adipose tissues axis and the RA of gut microbial population. In particular, CST prevents the imbalance of gut microbiota and restores the RA of Bacteroidetes/Firmicutes ratio in HFD-fed mice. It can be concluded that CST has potential anti-obesity effect that may work by suppression of obesity-promoting and induction of obesity-suppressing neuropeptides and adipokines that could modulate the RA of gut microbiota.

## 4. Materials and Methods

### 4.1. Animals and Diets

Six-week male C57BL/6J mice (Koatech, Seoul, South Korea) were housed in cages (one mouse per cage) and maintained under a 12/12 h light-dark cycle (09:00–21:00) at a constant temperature (22 ± 2 °C) and relative humidity (40%–60%). The animals were acclimatized in this condition for four weeks with free access to water and normal research diet (NRD; AIN-93G, Product # D10012G, Research Diets, Inc., New Brunswick, NJ, USA, *n* = 6) or high fat diet (HFD; Product # D12492, Research Diets, Inc., *n* = 18). The compositions of NRD (15.8 kcal% of energy was distributed in fat) and HFD (60 kcal % fat, Research Diets) are depicted in Supplementary Materials Table S1. The rationale behind the usage of AIN-93G as the matching control diet of HFD is the ability of this purified balanced diet formulation to serve as an ideal food for gestating and growing rodents. This diet is widely used along with HFD for research on obesity and weight gain [56,57]. After four weeks of acclimatization when a significant difference in the body weight between HFD-fed and NRD-fed mice was achieved, the HFD group was equally divided into three subgroups: HFD-fed only (HFD; *n* = 6), HFD-fed treated with orlistat (ORL; *n* = 6, a standard anti-obesity drug) and HFD-fed treated with CST (*n* = 6). The body weight and food intake of the animals were monitored weekly throughout the experimental period. Housing and care of the animals and all experimental steps were carried out per guidelines and the experimental protocol of the Research Animal Ethics Committee of Dongguk International Hospital, Dongguk University, Seoul, South Korea.

### 4.2. Preparation of CST

Powdered CST was purchased from Hanpoong Pharmaceutical (Daejeon, South Korea). The detail of CST formula consisting of 12 different herbs is depicted in supplementary Table S2. The herbs were mixed in 10 times volume of water, and subjected to decoction at 100 °C for 4 h. Following this, the extract was filtered and freeze dried for three to four days to obtain a dried final product without traces of water. Finally, the product was crushed into powdered form for use.

### 4.3. Treatments

The CST and ORL groups were fed by oral gavage with CST (700 mg/kg body weight) and ORL (10 mg/kg body weight), respectively, prepared in autoclaved distilled water. The mice in NRD and HFD groups were fed by oral gavage with distilled water only as vehicle. Drugs or vehicle were administered once a day for 12 weeks (six times per week). The ORL was purchased from Roche Pharmaceuticals (Roche, Nutley, NJ, USA).

### 4.4. Oral Glucose Tolerance Test (OGTT)

After ending the twelve-week treatment schedule, the mice were starved overnight for 16 h with free access to water prior to the OGTT, in accordance to the previous reports [58,59]. The OGTT was performed on the fasted animals by oral administration of glucose (prepared in autoclaved distilled water) at a dose of 1 g/Kg body weight. The glucose levels were measured at five different time points (0, 15, 30, 60, and 120 min) with the help of Accu-Check (Roche Diagnostics, Mannheim, Germany) using blood samples collected from the tail vein.

### 4.5. Samples Collection

Immediately after OGTT, the animals of all experimental groups were provided with their respective diets with free access to water for 8 h. They were then subjected to overnight fasting for 16 h prior to sampling, in compliance with the standard operating procedures for performing metabolic tests which describes that mice are normally fasted for either 14–18 h (overnight fast) or for 5–6 h (morning fast) in a typical metabolic study [60]. Following this, the animals were sacrificed under anesthesia induced by the intraperitoneal administration of a mixture of Zoletil^TM^ (Virbac, Carros, France) and Rompun^TM^ (Bayer, Leverkusen, Germany). After exsanguination, blood was collected quickly and allowed to clot for 30 min. The liver, hypothalamus, and adipose tissues (from kidney, testis, and intestine) were excised promptly, weighed and sectioned wherever applicable. The hypothalamus and adipose tissues samples for assessing the gene expression of obesity-related neuropeptides and adipokines, respectively were immediately submerged in RNAlater^®^ solution (Ambion, Austin, TX, USA) followed by snap freezing in liquid nitrogen and storage at −80 °C. Stool samples were collected in sterile tubes and stored at −80 °C for quantification of microbiota. Sera were separated from collected blood samples by centrifugation at 3000 rpm for 15 min at 4 °C and stored at −80 °C for further biochemical analyses. Liver and adipose tissue samples dedicated to histological analysis were fixed in 10% formalin and stored at room temperature until analyzed.

### 4.6. Serum Biochemistry Analyses

The level of fasting insulin in serum was measured using the enzyme-linked immunosorbent assay (ELISA) kit (Abcam, Cambridge, UK) following the kit manufacturer’s protocol. Other clinical markers such as total cholesterol (TC), triglyceride (TG), high-density lipoprotein (HDL), alanine transaminase (ALT) and aspartate aminotransferase (AST) were measured using the commercial kits (ASAN pharmaceutical, Seoul, South Korea) per manufacturer’s instructions.

### 4.7. Histological Analyses of Liver and Adipose Tissues

The FCS 22 Frozen Section Media (Leica Biosystem, Richmond, IL, USA)-embedded liver tissue samples for fat deposition measurement were sectioned at 5-μm thickness using a cryostat (CM1860, Leica Biosystem, Nussloch, Germany), then fixed on silicon-coated glass slides (Leica Biosystem). Sections were stained with oil-o-red solution (Cayman Chemical, Ann Arbor, MI, USA), then with hematoxylin (Merck, Darmstadt, Germany) counterstain. The paraffin-embedded liver and adipose tissue samples for analysis of the histological architecture of tissues were sectioned at 5-μm thickness using a microtome (RM2235, Leica Microsystems, Bannockburn, IL, USA) and fixed on silicon coated glass slides. Sections were stained with hematoxylin and eosin (Sigma Aldrich Inc., St. Louis, MO, USA). Stained slides were examined at 200× magnification under an inverted light microscope (BX61, Olympus, Tokyo, Japan) and the images were acquired using a digital camera (DP70, Olympus).

### 4.8. Analyses of Gene Expression of Neuropeptides and Adipokines

Total RNA from adipose tissue and hypothalamus samples was extracted using a commercial TRIzol^®^ reagent kit (Life Technologies, Carlsbad, CA, USA) according to the kit manufacturer’s instructions. The qualitative and quantitative analyses of the extracted RNA were performed by optical measurement at 260 nm and 280 nm using a spectrophotometer and gel electrophoresis on 1% agarose, respectively as described [61]. For cDNA synthesis, an equal quantity of each RNA sample (1 μg) was reverse transcribed using oligo-(dT) 18 cDNA RT PreMix kit (Bioneer, Daejeon, South Korea). The qRT-PCR was carried out on a Light Cycler 480^TM^ platform (Roche Applied Science) in a 96-well plate using a Light Cycler^®^ FastStart DNA Master SYBR Green kit (Roche Applied Science). The amplification reactions were performed in accordance with the kit manufacturer’s instructions in a total 20 μL volume of PCR mixture, containing 1 μL of cDNA, 10 pmole of each reverse and forward primers of a particular gene (Bioneer, Daejeon, South Korea; Table S3) 10 μL of SYBR green I master mix and 8 μL of nuclease free water. The conditions for PCR amplification reactions were as follows: an initial denaturation step at 95 °C for 10 min followed by 45 cycles of amplification encompassing denaturation at 95 °C for 10 s, annealing at 55–58 °C for 5 s and extension at 72 °C for 10 s. Following this reaction, a melting curve analysis was performed to verify the purity and specificity of the amplicon. All amplification reactions were carried out in duplicate and the gene expressions were normalized using glyceraldehyde-3-phosphatase dehydrogenase (*Gapdh*) as a housekeeping gene. The processing and analyses of the data were performed using a dedicated Light Cycler software (version 1.2, Roche Applied Science) supplied by the instrument manufacturer. The relative gene expressions were quantified following the standard 2^−∆Ct^ calculation, where C_t_ denotes the crossing threshold value calculated by the software and ∆C_t_ = (C_t-target gene_ − C_t-*Gapdh*)_.

### 4.9. Analyses of Fecal Microbial RA

Bacterial DNA was isolated from stored stool samples using stool DNA extraction kit (Quaigen, Korea Ltd., Seoul, South Korea). The quantitative and qualitative analyses of the extracted DNA samples were performed through the optical measurement at 260 nm and 280 nm using a Nanodrop spectrophotometer (Thermo Fisher Scientific, Waltham, MA, USA) and gel electrophoresis on 1% agarose. The qRT-PCR was carried out on a Light Cycler 480^TM^ system as mentioned above in a total 20 μL volume of PCR mixture containing 1 μL of stool DNA, 1 μL of primers (10 pmole each for reverse and forward primers), 10 μL of SYBR green I master mix, and 8 μL of nuclease free water. The PCR amplification was performed under the following conditions: pre-incubation at 94 °C for 15 s, primer annealing at 55 °C for 15 s, and elongation at 72 °C for 20 s. The melting curve was analyzed by heating the reaction mixture from 50 to 90 °C with a temperature transition time of 5 °C/s. All amplification reactions were carried out in duplicate, and the relative gene expressions were normalized with universal 16S rRNA primer. The details of the primers (Bioneer) are given in Table S4.

### 4.10. Statistical Analyses

For comparison between groups and significance analysis, one-way ANOVA followed by Least Significant Difference (LSD) post hoc test was used. The Statistical Package for Social Science software (SPSS Inc. Released 2008. SPSS Statistics for Windows, Version 17.0. SPSS Inc., Chicago, IL, USA) was employed for this purpose. PermutMatrix software (version 1.9.3 EN, Montpellier, France) was used for heatmap plots (Available at: http://www.lirmm.fr/∼caraux/PermutMatrix/). Data are presented as means ± SD (standard deviation). Different letters above bars indicate significantly different from each other at *p* < 0.05. Null hypotheses of no difference were rejected if *p*-values were less than 0.05.

## Supplementary Materials

Supplementary materials can be accessed at: http://www.mdpi.com/1420-3049/21/11/1522/s1.
