# Supplementary material for: CST, an Herbal Formula, Exerts Anti-Obesity Effects through Brain-Gut-Adipose Tissue Axis Modulation in High-Fat Diet Fed Mice
Source: Molecules. 2016 Nov 11;21(11):1522. doi: 10.3390/molecules21111522 (PMC6274029; doi:10.3390/molecules21111522)
Supplement: Supplementary file 1 [file molecules-21-01522-s001.pdf]

# Supplementary Materials: CST, a Herbal Formula, Exerts Anti-Obesity Effects through Brain-Gut-Adipose Tissue Axis Modulation in High-Fat Diet Fed Mice

**AbuZar Ansari, Shambhunath Bose, Mukesh Kumar Yadav, Jing-Hua Wang, Yun-Kyung Song, Seong-Gyu Ko and Hojun Kim**

**Table S1.** Details of diet composition.

| Normal Research Diet (NRD) | | | High Fat Diet (HFD) | | |
|---|---|---|---|---|---|
| **Product # D10012G** | **gm%** | **kcal%** | **Product # D12492** | **gm %** | **kcal %** |
| Protein | 20.0 | 20.3 | Protein | 26.2 | 20 |
| Carbohydrate | 64.0 | 63.9 | Carbohydrate | 26.3 | 20 |
| Fat | 7.0 | 15.8 | Fat | 34.9 | 60 |
| Total | | 100 | Total | | 100 |
| Kcal/gm | 3.9 | | Kcal/gm | 5.24 | |

**Table S2.** Details of CST composition.

| Pharmacognostic Name | Dried Weight (g) |
|---|---|
| Coicis Semen | 3.75 g |
| Castaneae Semen | 3.75 g |
| Raphani Semen | 1.875 g |
| Ephedrae Herba | 1.25 g |
| Platycodi Radix | 1.25 g |
| Liriopis Tuber | 1.25 g |
| Schizandrae Fructus | 1.25 g |
| Acori Graminei Rhizoma | 1.25 g |
| Polygalae Radix | 1.25 g |
| Asparagi Radix | 1.25 g |
| Zizyphi Spinosae Semen | 1.25 g |
| Longanae Arillus | 1.25 g |

**Table S3.** Details of neuropeptides and adipokines primer sequences.

| Gene | Forward Primer Sequence | Reverse Primer Sequence | Reference |
|---|---|---|---|
| *Agrp* | 5′-CGGAGGTGCTAGATCCACAGA-3′ | 5′-AGGACTCGTGCAGCCTTACAC-3′ | [60] |
| *Npy* | 5′-TACTCCGCTCTGCGACACTACA-3′ | 5′-AATCAGTGTCTCAGGGCTGGAT-3′ | [61] |
| *Cart* | 5′-CTGCAATTCTTTCCTCTTGAAGTG-3′ | 5′-GGGAATATGGGAACCGAAGGT-3′ | [61] |
| *Pomc* | 5′-TGCTTCAGACCTCCATAGATGTGT-3 | 5′-GGATGCAAGCCAGCAGGTT-3′ | [61] |
| *Adipoq* | 5′-TGTTCCTCTTAATCCTGCCCA-3′ | 5′-CCAACCTGCACAAGTTCCCTT-3′ | [62] |
| *Adn* | 5′-GCAGTGGGTGCTCAGTGCT-3′ | 5′-TCGTCATCCGTCACTCCATC-3′ | [63] |
| *Gapdh* | 5′-GTCACCAGGGCTGCCTTCT-3′ | 5′-CATTGAACTTGCCGTGGGTA-3′ | [63] |
| *Lep* | 5′- CTGCCCCCCAGTTTGATG -3′ | 5′- GCCAGGCTGCCAGAATTG -3′ | [64] |
| *Lcn2* | 5′-TGCCACTCCATCTTTCCTGTT-3′ | 5′-GGGAGTGCTGGCCAAATAAG-3′ | [65] |
| *Rbp4* | 5′-ACTG GGGTGTAGCCTCCTTT-3′ | 5′-GGTGTCGTAGTCCGTGTCG-3′ | [66] |
| *Vasp* | 5′-CCTGTGGCGTTAGAGTA-3′ | 5′-GGCGGAAAGAGGTGATT-3′ | [67] |
| *Visf* | 5′-ACATAGGACACCAGCG-3′ | 5′-AAACACGAACCCACAC-3′ | [67] |
| *Retn* | 5′-GTACCCACGGGATGAAGAACC-3′ | 5′-CAAGGTTGGATGGGGGCGAAGGG-3 | [68] |

**Table S4.** Details of gut microbiota primer sequences.

| Bacteria | Forward Primer Sequence | Reverse Primer Sequence | Ref. |
|---|---|---|---|
| *Lactobacillus* | 5′-GAGGCAGCAGTAGGGAATCTTC-3′ | 5′-GGCCAGTTACTACCTCTATCCTTCTTC-3′ | [26] |
| Bacteroidetes | 5′-GGARCATGTGGTTTAATTCGATGAT-3′ | 5′-AGCTGACGACAACCATGCAG-3′ | [69] |
| Fermicutes | 5′-GGAGYATGTGGTTTAATTCGAAGCA-3′ | 5′-AGCTGACGACAACCATGCAC-3′ | [69] |
| *Akkermansia* | 5′-CAGCACGTGAAGGTGGGGAC-3′ | 5′-AGCTGACGACAACCATGCAG-3′ | [70] |
| *Bacteroides* | 5′-GAAGGTCCCCCACATTG-3′ | 5′-CGCKACTTGGCTGGTTCAG-3′ | [71] |
| *Prevotella* | 5′-CACRGTAAACGATGGATGCC-3′ | 5′-GGTCGGGTTGCAGACC-3′ | [71] |
| *Roseburia* | 5′-GATGAAGTATCTCGGTATGT-3′ | 5′-CTACGCTCCCTTTACAC-3′ | [71] |
| *Bifidobacterium* | 5′-CGCGTCTGGTGTCAAAG-3″ | 5′-CCCCACATCCAGCATCCA-3′ | [72] |
| *Ruminococcus* | 5′-GGCGGCCTACTGGGCTTT-3′ | 5′-CCAGGTGGATAACTTATTGTGTTAA-3′ | [73] |
| 16sRNA universal primers | 27F, 5′-AGAGTTTGATCCTGGCTCAG-3′ | 1525R, 5′-AAGGAGGTGATCCAGCC-3′ | [74] |

## References


60. Knauf, C.; Rieusset, J.; Foretz, M.; Cani, P.D.; Uldry, M.; Hosokawa, M.; Martinez, E.; Bringart, M.; Waget, A.; Kersten, S. Peroxisome proliferator-activated receptor-α-null mice have increased white adipose tissue glucose utilization, GLUT4, and fat mass: role in liver and brain. *Endocrinology* **2006**, *147*, 4067–4078.
61. Schéle, E.; Grahnemo, L.; Anesten, F.; Hallén, A.; Bäckhed, F.; Jansson, J.-O. The gut microbiota reduces leptin sensitivity and the expression of the obesity-suppressing neuropeptides proglucagon (Gcg) and brain-derived neurotrophic factor (Bdnf) in the central nervous system. *Endocrinology* **2013**, *154*, 3643–3651.
62. Liu, Y.; Zhang, Y.-D.; Guo, L.; Huang, H.-Y.; Zhu, H.; Huang, J.-X.; Liu, Y.; Zhou, S.-R.; Dang, Y.-J.; Li, X. Protein inhibitor of activated STAT 1 (PIAS1) is identified as the SUMO E3 ligase of CCAAT/ enhancer-binding protein β (C/EBPβ) during adipogenesis. *Mol. Cell. Biol.* **2013**, *33*, 4606–4617.
63. Li, R.; Song, H.; Shi, W.; Hu, S.; Yang, Y.; Tang, J.; Chen, M.; Chen, J. Galanin inhibits leptin expression and secretion in rat adipose tissue and 3T3-L1 adipocytes. *J. Mol. Endocrinol.* **2004**, *33*, 11–19.
64. Qu, Y.; Zhang, Q.; Ma, S.; Liu, S.; Chen, Z.; Mo, Z.; You, Z. Interleukin-17A Differentially Induces Inflammatory and Metabolic Gene Expression in the Adipose Tissues of Lean and Obese Mice. *Int. J. Mol. Sci.* **2016**, *17*, 522.
65. Zhang, J.; Wu, Y.; Zhang, Y.; LeRoith, D.; Bernlohr, D.A.; Chen, X. The role of lipocalin 2 in the regulation of inflammation in adipocytes and macrophages. *Mol. Endocrinol.* **2008**, *22*, 1416–1426.
66. Mohapatra, J.; Sharma, M.; Acharya, A.; Pandya, G.; Chatterjee, A.; Balaraman, R.; Jain, M.R. Retinol-binding protein 4: a possible role in cardiovascular complications. *Br. J. Pharmacol.* **2011**, *164*, 1939–1948.
67. Liu, J.; Wang, L.; Zhang, A.; Di, W.; Zhang, X.; Wu, L.; Yu, J.; Zha, J.; Lv, S.; Cheng, P. Adipose tissue-targeted 11. BETA.-hydroxysteroid dehydrogenase type 1 inhibitor protects against diet-induced obesity. *Endocr. J.* **2011**, *58*, 199–209.
68. Sun, J.; Xu, Y.; Deng, H.; Sun, S.; Dai, Z.; Sun, Y. Intermittent high glucose exacerbates the aberrant production of adiponectin and resistin through mitochondrial superoxide overproduction in adipocytes. *J. Mol. Endocrinol.* **2010**, *44*, 179–185.
69. Guo, X.; Xia, X.; Tang, R.; Zhou, J.; Zhao, H.; Wang, K. Development of a real-time PCR method for Firmicutes and Bacteroidetes in faeces and its application to quantify intestinal population of obese and lean pigs. *Lett. Appl. Microbiol.* **2008**, *47*, 367–373.
70. Everard, A.; Belzer, C.; Geurts, L.; Ouwerkerk, J.P.; Druart, C.; Bindels, L.B.; Guiot, Y.; Derrien, M.; Muccioli, G.G.; Delzenne, N.M. Cross-talk between Akkermansia muciniphila and intestinal epithelium controls diet-induced obesity. *Proc. Nat. Acad. Sci. USA* **2013**, *110*, 9066–9071.
71. Song, M.-Y.; Wang, J.-H.; Eom, T.; Kim, H. Schisandra chinensis fruit modulates the gut microbiota composition in association with metabolic markers in obese women: A randomized, double-blind placebo-controlled study. *Nutr. Res.* **2015**, *35*, 655–663.
72. Delroisse, J.-M.; Boulvin, A.-L.; Parmentier, I.; Dauphin, R.D.; Vandenbol, M.; Portetelle, D. Quantification of Bifidobacterium spp. and Lactobacillus spp. in rat fecal samples by real-time PCR. *Microbiol. Res.* **2008**, *163*, 663–670.
73. Ramirez-Farias, C.; Slezak, K.; Fuller, Z.; Duncan, A.; Holtrop, G.; Louis, P. Effect of inulin on the human gut microbiota: stimulation of Bifidobacterium adolescentis and Faecalibacterium prausnitzii. *Bri. J. Nutr.* **2009**, *101*, 541–550.
74. Wang, J.-H.; Bose, S.; Kim, H.-G.; Han, K.-S.; Kim, H. Fermented Rhizoma Atractylodis Macrocephalae alleviates high fat diet-induced obesity in association with regulation of intestinal permeability and microbiota in rats. *Sci. Rep.* **2015**, *5*, doi:10.1038/srep08391.
